# Research on the Effect of Oxygen Ions on the Coordination Structure and Electrochemical Behavior of Titanium Ions in NaCl-KCl Melt

**DOI:** 10.3390/ma18133161

**Published:** 2025-07-03

**Authors:** Shaolong Li, Peizhu Mao, Tianzhu Mu, Fuxing Zhu, Shengwei Li

**Affiliations:** 1 State Key Laboratory of Vanadium and Titanium Resourcces Comprehensive Utilization, Airport Road 10, Panzhihua 617099, China; 13237641651@163.com (T.M.); 15205199180@163.com (F.Z.); 2 School of Material Science and Engineering, Zhengzhou University, Science Road 100, Zhengzhou 450001, China; mpz12345678@gs.zzu.edu.cn; 3 Western Mining Group Co., Ltd., Xining 810000, China

**Keywords:** Titanium ions, molten salt, oxygen ions, electrochemical behavior, coordination relationship

## Abstract

Presently, extensive research has been conducted on the electrochemical behavior of titanium ions in molten salt, especially in relation to titanium fluoride coordination. However, there is limited research on the coordination between titanium and oxygen. Consequently, this research delved into the influence of oxygen ions on the electrochemical behavior and coordination properties of titanium ions through the utilization of both electrochemical and spectroscopy techniques. The study involved the use of cyclic voltammetry (CV), square wave voltammetry (SWV), and the open-circuit potential (OCP) method to explore the electrochemical properties of titanium ions at different titanium-oxygen ratios. Furthermore, X-ray photoelectron spectroscopy (XPS) and Raman spectroscopy were applied to assess the presence of titanium ions in molten salt and the coordination structure of titanium ions and anions in molten salts, respectively. The results demonstrate that with an increase in oxygen ion content, chloride ions are gradually replaced by oxygen ions, forming TiO*_x_*Cl*_y_^m^^−^* complexes.

## 1. Introduction

The presence of electrolyte constituents in molten salt is critical for maintaining the stability of titanium ions. Extensive research has been conducted on various molten salt electrolytes using chloride molten salts, fluoride molten salts, and chloride-fluoride molten salts as electrolytes [1,2,3,4,5,6,7]. Titanium ions exist in multiple valence states. Disproportionation or neutralization reactions may occur between different valence states, and their equilibrium relationships can be described by the following reactions:

3Ti^2+^ = Ti + 2Ti^3+^ (1)

4Ti^3+^ = Ti + 3Ti^4+^ (2)

In molten salt systems, titanium ions can exist in three distinct valence states: Ti^2+^, Ti^3+^, and Ti^4+^ [8,9,10,11]. In the chloride molten salt system with Ti^3+^, it is generally reduced in two steps: Ti^3+^ → Ti^2+^ and Ti^2+^ → Ti [12,13]. In the fluoride molten salt system, Ti^3+^ is directly reduced to titanium [3,14]. In the chloride–fluoride molten salt system, an intermediate reduction step is involved under low fluoride concentration conditions, while titanium ions are obtained through a one-step reduction when the fluoride concentration reaches a certain value [12,15,16]. This phenomenon arises from the introduction of a specific quantity of fluoride ions into the electrolyte, leading to the disproportionation reaction of Ti^3+^ and disrupting the equilibrium of the reaction. The complexes resulting from the interaction of high-valent cations and fluoride ions exhibit increased stability. Furthermore, fluoride ions, due to their smaller radius compared to chloride ions, are more inclined to establish coordination bonds with cations in molten salts [11,17]. Consequently, when the fluoride ion concentration reaches a certain level, Ti^2+^ will no longer be present, and the ions in equilibrium with titanium metal will be Ti^3+^ and Ti^4+^. Some studies also indicate that the form of titanium ions varies in different compositions of electrolytes. In alkaline chloride melts, Ti^2+^ exists in a more stable form, while in alkaline fluoride melts, Ti^3+^ and Ti^4+^ are the main forms. In most chloride melts, there is an equilibrium between Ti^2+^, Ti^3+^, and metallic titanium, which can be represented by reaction (1).

In chloride molten salt systems, three different types of coordinated titanium ions are present: TiCl_6_^2−^, TiCl_6_^3−^, and TiCl_6_^4−^ [18]. In molten salt, oxygen ions are often difficult to completely remove. When there is a certain amount of oxygen ions in the molten salt, the coordination form between titanium ions and chloride ions changes. Compared to chloride ions, oxygen ions have a smaller ionic radius and are more likely to form coordination relationships with cations in the molten salt. As the oxygen ion content increases, the chloride ions will gradually be replaced by oxygen ions, forming TiO*_x_*Cl*_y_^m^*^−^ complexes [19,20]. When the oxygen content reaches a certain value, the chloride ions will be completely replaced by oxygen ions. It can be seen that the coordination structure of titanium ions in the chloride–oxygen system is complex and diverse. Kaai Okada et al. [21] indicated that the dissociated oxygen atoms within the molten substance could potentially interact significantly with titanium ions and exhibit a tendency towards coordination. It is theorized that as the concentration of oxygen ions increases, the coordination number of oxygen atoms also increases [17,22,23].

Obviously, the coordination relationship between oxygen ions and titanium ions is closely related to the electrochemical behavior of titanium ions, which in turn affects the efficiency of electrodeposition and product quality. At present, research on the coordination behavior of titanium ions and oxygen ions is still unclear. Herein, this paper systematically investigates the electrochemical behavior and coordination structure of titanium ions under different oxygen ion contents, revealing the relationship between them, which is of significant importance for the selection and purification of electrolytes.

## 2. Experimental

In this study, a eutectic mixture of NaCl-KCl was chosen as the electrolyte (NaCl, purity 99.8%; KCl, purity 99.8%, purchased from Macklin Biochemical Co. Ltd., Shanghai, China). A total of 100 g of NaCl-KCl salt (with a molar ratio of 1:1) was weighed and uniformly mixed before being placed into a corundum crucible with an inner diameter of 65 mm. Under an argon atmosphere, the temperature was raised to 300 °C and maintained at this level for 2 h to eliminate moisture from the salt. Subsequently, the temperature was further increased to 750 °C to carry out electrochemical tests.

To investigate the electrochemical behavior of titanium ions in NaCl-KCl molten salt systems with different molten salt compositions, an amount of TiCl_2_ (0.25 wt.%) was added to the NaCl-KCl molten salt. This paper intends to introduce oxygen ions through two different approaches. One method involves conducting electrolysis using an oxygen-containing anode, which is relatively similar to the actual electrolysis process. However, it is challenging to control the actual concentration of oxygen ions. The anode used in this experiment was prepared in our laboratory via the carbothermal reduction method. This method employed titanium dioxide (TiO_2_) and graphite powder (C) as raw materials. After thorough mixing, the mixture was pressed into round tablets with a diameter of 12 mm and a thickness of 1.5 mm. These tablets were then held at 1600 °C for 4 h under an argon atmosphere to obtain the soluble anode, TiC*_x_*O*_y_*, with different carbon to oxygen ratios. To obtain TiC_0.7_O_0.3_, TiC_0.6_O_0.4_, TiC_0.5_O_0.5_, TiC_0.4_O_0.6_, and TiC_0.3_O_0.7_, the theoretical molar ratios of TiO_2_ to graphite should be 1:2.4, 1:2.2, 1:2.0, 1:1.8, and 1:1.6, respectively. The other method involves introducing oxygen ions in the form of Na_2_O, which allows for better quantification. The correlation between the mass of added Na_2_O and the molar ratio of O^2−^/Ti^2+^ is shown in Table 1. To ensure the complete dissolution of Na_2_O and TiCl_2_, the mixture was left to stand for 0.5 h after the addition of reagents before conducting the test.

**Figure 1:**
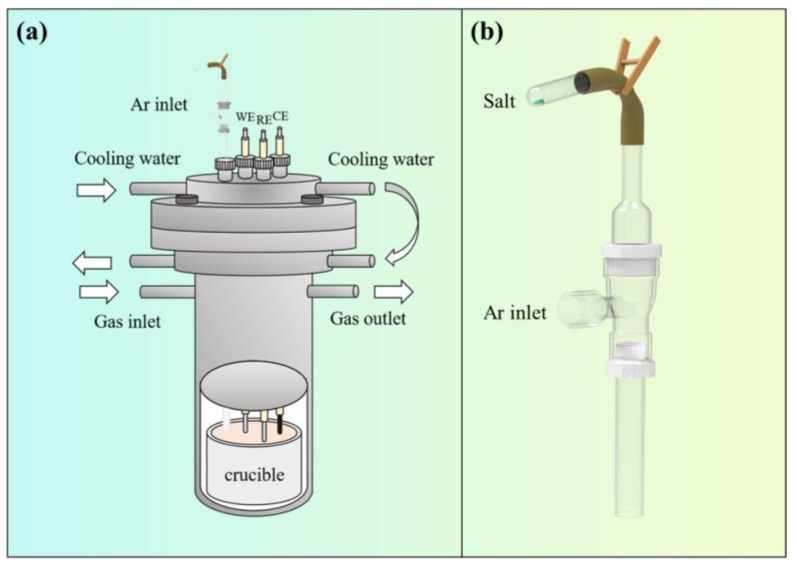
Schematic diagram of electrolysis device. (**a**) Electrolysis furnace; (**b**) feeding device.

Figure 1 illustrates a schematic diagram of the online feeding device. Prior to feeding, TiCl_2_ was placed into the test tube shown in Figure 1b and connected to a rubber tube; the fixture was opened, salt was poured into the empty cavity of the feeding device, and it was carried into the crucible by flowing argon gas. This feeding method helps reduce the amount of air mixed in during the feeding process.

In this experiment, all electrochemical tests were recorded with the Nova 2.1 software package controlled by AutoLab PGSTAT 302 N (Metrohm Autolab B.V., Utrecht, The Netherlands). During the electrolysis process, TiC*_x_*O*_y_* was utilized as the anode and a molybdenum rod as the cathode. After the electrolysis, the salt was quickly taken and quenched for X-ray Photoelectron Spectroscopy (XPS) testing to evaluate the valence state of titanium ions. The electrochemical behaviors of titanium ions in NaCl-KCl melts were analyzed using electrochemical testing techniques, including cyclic voltammetry (CV), square wave voltammetry (SWV), linear sweep voltammetry (LSV), and open-circuit potential (OCP). Electrochemical tests were conducted in a three-electrode system. The working electrode was a glassy carbon electrode with a diameter of 2.8 mm, the reference electrode was a platinum wire electrode with a diameter of 2.0 mm, and the counter electrode was a graphite rod with a diameter of 6.0 mm. Prior to use, all electrodes were meticulously polished and cleaned using ultrasonic techniques. Immediately after the electrochemical examination, Raman spectroscopy was performed on the salt to investigate the coordination bond between titanium and oxygen ions.

## 3. Results and Discussion

### 3.1. Characterization of Oxygen

**Figure 2:**
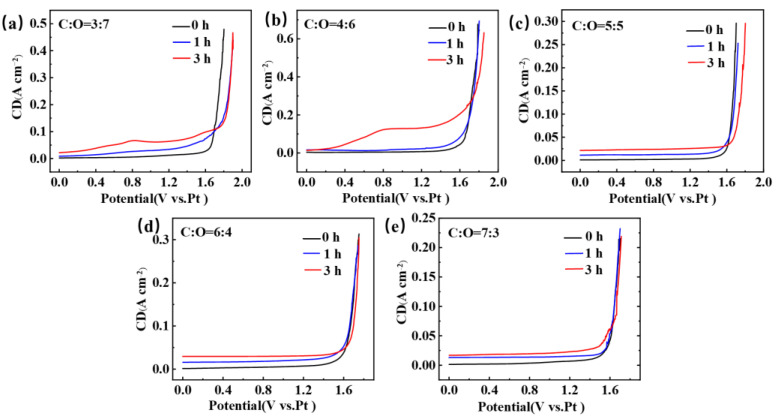
The LSV curves obtained after electrolysis of TiC*_x_*O*_y_* anodes for 0 h, 1 h, and 3 h in a NaCl-KCl eutectic molten salt at 750 °C. (**a**) C to O ratio is 3 to 7; (**b**) C to O ratio is 4 to 6; (**c**) C to O ratio is 5 to 5; (**d**) C to O ratio is 6 to 4; (**e**) C to O ratio is 7 to 3.

Figure 2 shows the LSV obtained after electrolysis of TiC*_x_*O*_y_* anodes with different carbon-to-oxygen ratios in NaCl-KCl molten salt, with glassy carbon as the working electrode. During the processes of electrolysis and electrochemical testing, we followed a specific procedure to ensure that we could obtain meaningful LSV data. Before initiating the electrolysis process, we first recorded the LSV curve of the electrode in the NaCl-KCl molten salt with a standard three-electrode system. After obtaining the pre-electrolysis LSV curve, we commenced the electrolysis process. A two-electrode system was employed during electrolysis, and the electrolysis was carried out at a constant potential of 2.8 V for 1 h or 3 h. Once the designated electrolysis time (1 h or 3 h) had elapsed, we stopped the electrolysis and utilized a three-electrode system to conduct LSV measurements. The LSV measurement after electrolysis was performed under the same conditions as the pre-electrolysis measurement, including the same potential range, scan rate, and electrolyte composition. This allowed us to directly compare the pre-and post-electrolysis LSV curves. It can be observed that the LSV obtained under five different carbon-to-oxygen ratio conditions all exhibit a sharp increase in current near 1.60 V, which is due to the decomposition of NaCl and the evolution of chlorine gas [24,25]. However, the difference lies in the fact that TiC_0.7_O_0.3_, TiC_0.6_O_0.4_, and TiC_0.5_O_0.5_ exhibit a smooth LSV curve after 1 h and 3 h of electrolysis, with no peaks appearing and no significant changes compared to the curve before electrolysis. But, TiC_0.4_O_0.6_ and TiC_0.3_O_0.7_ show significant changes in the LSV curve after 1 h and 3 h of electrolysis compared to before electrolysis. A distinct small peak appears around 0.8 V, and the chlorine gas evolution potential shifts towards positive. Furthermore, with prolonged electrolysis time, the peak near 0.8 V becomes more pronounced. The appearance of this peak may be due to the incomplete combination of carbon and oxygen into CO during the electrolysis process of TiC*_x_*O*_y_*, and the remaining oxygen ions entering the molten salt and being oxidized on the surface of the working electrode. As the electrolysis time prolongs, the concentration of oxygen ions increases.

**Figure 3:**
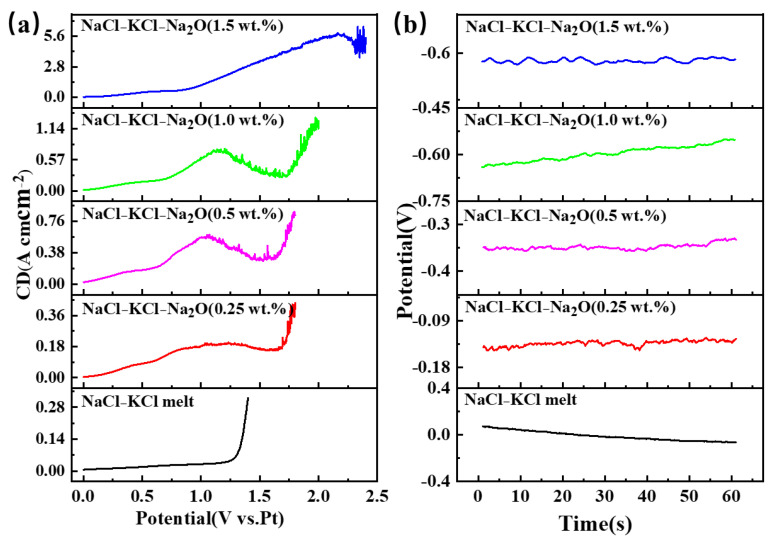
The LSV and OCP curves in the NaCl-KCl-Na_2_O molten salt with different molar ratios of oxygen ions to titanium ions. (**a**) LSV curves; (**b**) OCP curves.

Due to the inability to precisely regulate the oxygen ion concentration in the molten salt during electrolysis, sodium oxide (Na_2_O) was added in Figure 3 to control the oxygen ion content within the system. In Figure 3a, it can be observed that the LSV curve obtained in NaCl-KCl molten salt without Na_2_O added is a smooth curve. After adding 0.25 wt.% Na_2_O, a clear oxygen ion oxidation signal appeared around 1.0 V. As the oxygen content increases, the peak intensity shows an increasing trend, with the potential gradually shifting towards the positive direction. When the oxygen content reaches 1.5 wt.%, both the peak intensity and potential undergo significant changes. Figure 3b displays the trend of the open circuit potential under varying oxygen contents in NaCl-KCl-Na_2_O molten salt. There is a noticeable shift towards negativity as the oxygen content increases, with the open circuit potential rising from −0.062 V to −0.634 V.

**Figure 4:**
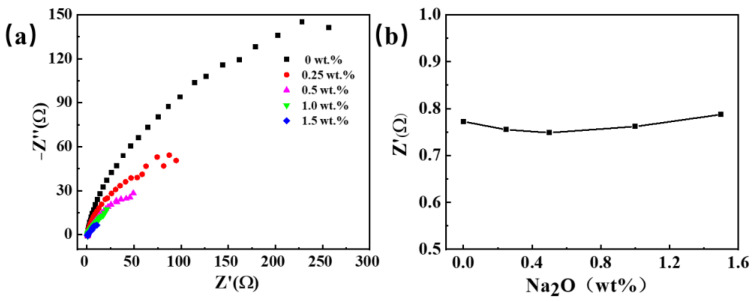
The electrochemical impedance spectra were obtained by adding different sodium oxides to the NaCl-KCl molten salt. (**a**) EIS curves; (**b**) electrolyte resistance at different Na_2_O concentrations.

Figure 4 illustrates electrochemical impedance spectroscopy (EIS) at varying concentrations of oxygen ions in the salt, examining the impact of oxygen ion incorporation on resistance alteration. The findings indicate a decrease in electrolyte resistance initially, followed by a gradual increase with rising oxygen content. Nevertheless, due to its small variation, it can be considered that a restricted influence of oxygen ion introduction on the electrolyte’s conductivity.

### 3.2. Determination of the Valence State of Titanium Ions

**Figure 5:**
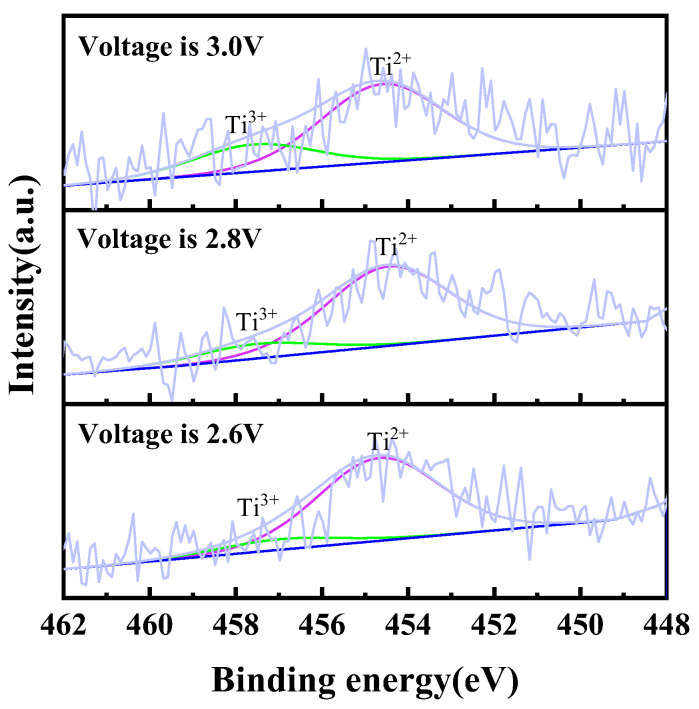
Ti 2p XPS spectra of electrochemical dissolution of titanium ions by using TiC*_x_*O*_y_* as anode at different voltages in NaCl-KCl molten salt at 750 °C.

In order to confirm the existence of titanium ions in the chloride molten salt system, electrolysis was performed with TiC_0.5_O_0.5_ serving as the anode and a molybdenum rod as the cathode in NaCl-KCl molten salt under different voltages (2.6 V, 2.8 V, and 3.0 V) over a period of 2 h. Post-electrolysis, salt was employed for sample preparation, followed by the execution of an XPS test. To ensure greater accuracy in the test structure, it is imperative to raise the concentration of titanium ions in the molten salt. This can be achieved by increasing the mass of the anode TiC_0.5_O_0.5_ and decreasing the mass of the molten salt during the experiment. Upon completion of electrolysis, it is necessary to promptly extract the salt and rapidly cool the solution in order to minimize the disproportionation of intermediate valence titanium ions. The findings of the experiment can be observed in Figure 5. The above results indicate that with the increase in electrolysis voltage, the content of trivalent titanium ions in the electrolyte increases, while the content of divalent titanium ions decreases. The occurrence of this phenomenon is linked to the electrolysis procedure. The rise in electrolysis voltage during the electrolysis process contributes to a stronger driving force for the oxidation of titanium at the anode, ultimately resulting in an increased proportion of high-valence titanium ions, a similar phenomenon reported in some literature [26]. The information presented above suggests that when TiC_0.5_O_0.5_ is used as the anode for electrolysis, the titanium ions entering the molten salt exist in a mixed valence state, including both divalent and trivalent states.

### 3.3. Reduction of TiCl_x_ in Molten NaCl–KCl Under Various Titanium-Oxygen Ratios

**Figure 6:**
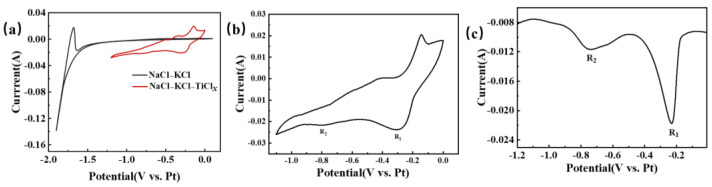
The electrochemical testing curve of titanium ions in NaCl-KCl molten salt is illustrated. The temperature is set at 750 °C, with a glassy carbon working electrode of 2.8 mm diameter, a platinum wire reference electrode of 1 mm diameter, and a graphite rod counter electrode of 6 mm diameter. (**a**) CV curve before and after the addition of TiCl_2_. scan rate: 100 mVs^−1^; (**b**) CV curve in the NaCl-KCl-TiCl*_x_* system, scan rate: 100 mVs^−1^, potential range: 0~−1.1 V; (**c**) SWV curve in the NaCl-KCl-TiCl*_x_* system, frequency: 25 Hz, potential range: 0~−1.2 V.

Figure 6 provides a visual representation of the electrochemical testing curve for titanium ions in NaCl-KCl molten salt. In Figure 6a, the CV test in NaCl-KCl blank molten salt is illustrated by the black curve. The potential undergoes a scan from 0 V to −1.5 V, with no notable change in current. The background current fluctuation remains within 1 mA, indicating the absence of any reaction in this potential range. However, as the negative scan continues, the current experiences a significant change, exhibiting a sharp increase. This change corresponds to the precipitation of alkali metal sodium [24,27,28,29]. Under the condition of maintaining constant parameters, a subsequent test was performed by introducing TiCl_2_ into the NaCl-KCl molten salt. The resulting CV curve, depicted as the red curve in Figure 6a, displayed distinct fluctuations between 0 and −1.1 V. The peaks observed during the negative scan corresponded to the reduction signal of ions, whereas the peaks observed during the positive scan corresponded to the oxidation signal of either ions or metals. Hence, it can be inferred that a conspicuous redox reaction took place following the introduction of titanium ions into the NaCl-KCl molten salt. To facilitate a more direct observation of the reduction process of titanium ions, CV and SWV tests were repeated within a relatively limited potential range. The obtained curves are illustrated in Figure 6b,c. On the CV curve, the reduction peak signals R_1_ and R_2_ can be easily discerned at −0.3 V and −0.79 V, respectively. These reduction peaks are also clearly observable on the SWV graph. According to references [12,13], R_1_ and R_2_ correspond to Ti^3+^ → Ti^2+^ and Ti^2+^ → Ti, respectively.

**Figure 7:**
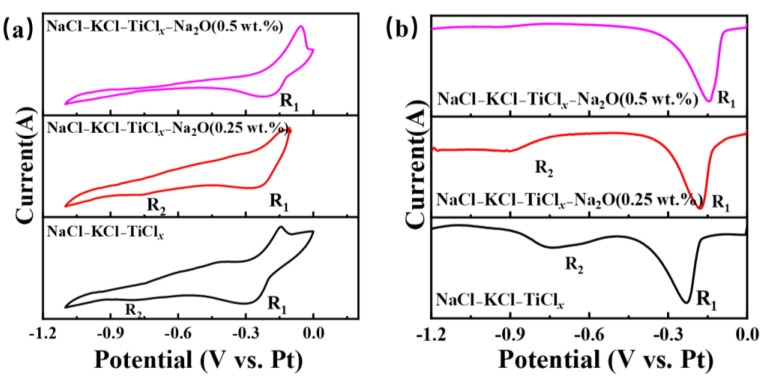
The electrochemical testing curves of different oxygen ions and titanium ions in the molten salt of NaCl-KCl-TiCl_2_-Na_2_O are depicted. (**a**) Shows the cyclic voltammetry (CV) curve at a scan rate of 100 mV/s, and (**b**) displays the square wave voltammetry (SWV) curve at a frequency of 25 Hz.

Further investigation was conducted on the impact of the electrochemical reduction behavior of titanium ions in the NaCl-KCl-TiCl_2_ system following the introduction of Na_2_O in Figure 7. Figure 7a shows the CV curve of titanium ions in the NaCl-KCl-TiCl_2_ molten salt system at different Na_2_O concentrations. When the oxygen content is 0.25 wt.%, the reduction peaks R_1_ and R_2_ become noticeably weaker. As the oxygen content continues to increase, the R_2_ peak disappears, and although the peak intensity of R_1_ shows no significant change, its potential shifts towards the positive direction. Furthermore, SWV testing was performed under identical conditions, and the corresponding SWV curve is depicted in Figure 7b. The R_2_ peaks disappear when the oxygen concentration is at 0.5 wt.%. The outcome demonstrates that the electrochemical reduction behavior of titanium ions undergoes significant changes upon the introduction of oxygen ions into the molten salt. The distinct polarization forces exhibited by the anion and cation in the electrolyte contribute to the modification of the metal ions’ morphology and potential intermediate valence states. This alteration subsequently impacts the electrochemical reduction pathway of the metal ions [17].

### 3.4. Titanium-Oxygen Coordination Behavior

**Figure 8:**
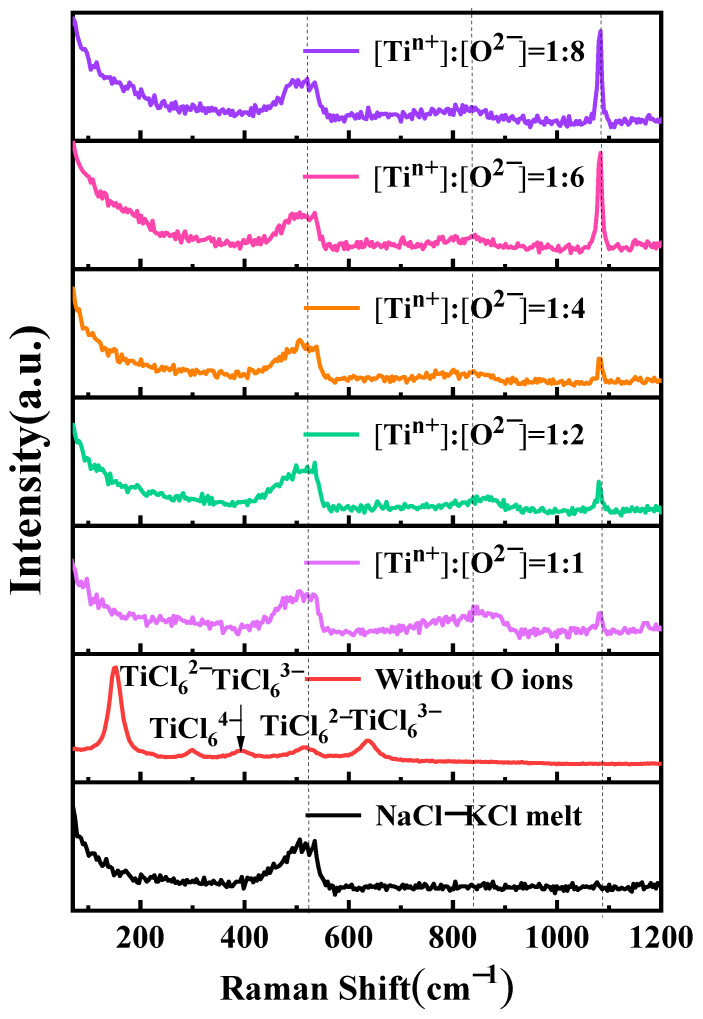
Raman test curves for different oxygen ion to titanium ion molar ratios in the molten salt of NaCl-KCl-TiCl_2_-Na_2_O.

Figure 8 shows the impact of oxygen ions on the coordination structure of titanium ions. Samples were prepared through rapid cooling using in-situ salt extraction for Raman spectroscopy analysis of NaCl-KCl-TiCl_2_-Na_2_O salts at varying Na_2_O concentrations. A distinct peak is observed at 149 cm^−1^ in the NaCl-KCl-TiCl_2_ salt, corresponding to TiCl_6_^2−^ [18,30]. Additionally, four smaller peaks are observed at 296 cm^−1^, 391 cm^−1^, 514 cm^−1^, and 635 cm^−1^, corresponding to TiCl_6_^4−^, TiCl_6_^3−^, TiCl_6_^2−^, and TiCl_6_^3−^, respectively [18]. Upon the introduction of oxygen ions, with a ratio of *n*(Ti^2+^):*n*(O^2−^) of 1:1 in the molten salt, new small peaks appear at 855 cm^−1^ and 1083 cm^−1^. This is attributed to the alteration of the titanium-chlorine coordination structure due to the presence of oxygen ions, resulting in the formation of a novel titanium-oxygen-chlorine complex. As the oxygen ion content increases, the peak at 855 cm^−1^ exhibits a diminishing trend. However, when the ratio of *n*(Ti^2+^):*n*(O^2−^) is 1:6, the peak at this position demonstrates an increasing trend. The peak at 1083 cm^−1^ consistently displays an increasing trend with an increase in oxygen content, and a sharp rise is observed when the ratio of *n*(Ti^2+^):*n*(O^2−^) is 1:6.

The reason behind the aforementioned phenomenon is the presence of titanium ions in the form of complexes in molten chloride salt. Due to the existence of titanium ions in different oxidation states, disproportionation reactions occur in the intermediate oxidation state of titanium ions [10]. When a certain amount of oxygen ions is introduced into the electrolyte, displacement reactions take place with chloride ions. This is because oxygen ions, having a smaller ionic radius compared to chloride ions, can readily replace chloride ions and establish coordination relationships with titanium ions in the molten salt. Consequently, in molten salts containing titanium ions, oxygen ions form coordination compounds with titanium. The formation of these complexes leads to a change in the equilibrium of titanium ions. Raman spectroscopy analysis effectively reveals this phenomenon.

## 4. Conclusions

In this study, the electrochemical behavior of titanium ions in various electrolyte solutions and the coordination structure relationship between titanium ions and oxygen ions were examined using electrochemical analysis and spectroscopic analysis. Based on the findings from these experiments, the following conclusions can be deduced:

(1) The molten salt underwent an LSV test after the electrolysis of TiC*_x_*O*_y_* with different ratios. The results demonstrated that for C/O ratios of 7/3, 6/4, and 5/5, there was an absence of oxygen ion infiltration into the electrolyte during the electrolysis process. In contrast, when the C/O ratios were adjusted to 4/6 or 3/7, oxygen ions were found to enter the electrolyte.
(2) Investigation on the electrochemical behavior of titanium ions under different oxygen ion conditions using CV and SWV. An increase in oxygen ion content led to noticeable changes in both peak intensity and potential on the LSV curve, along with a significant negative shift in the open-circuit potential. In the NaCl-KCl-TiCl_2_ molten salt, trivalent titanium ions are reduced to metallic titanium through two steps: Ti(III)-Ti(II) and Ti(II)-Ti. The reduction steps of titanium ions have been decreased by adding oxide ions into the molten salt.
(3) The XPS analysis in the NaCl-KCl-TiCl_2_ molten salt system demonstrates that the incorporation of oxygen ions gradually replaces the Ti-Cl bonds with Ti-O bonds. This substitution process results in the creation of new titanium chloro-oxygen complexes. The presence of TiO*_x_*Cl*_y_^m^^−^* is confirmed by both electrochemical and Raman tests.

## Figures and Tables

**Table 1:** Relationship between the mass of added Na_2_O and the molar ratio of O^2−^/Ti^2+^.

The Relationship Between the Molar Ratio of O^2−^/Ti^2+^	Theoretical Mass of Na_2_O Added (g)	Actual Mass of Na_2_O Added (g)
1:1	0.13	0.14
2:1	0.26	0.27
4:1	0.52	0.53
6:1	0.78	0.80
8:1	1.04	1.06

## Data Availability

The original contributions presented in the study are included in the article; further inquiries can be directed to the corresponding authors.
